# 21st Century Good Neighbor Program: An Easily Generalizable Program to Reduce Social Isolation in Older Adults

**DOI:** 10.3389/fpubh.2021.766706

**Published:** 2021-12-20

**Authors:** Shashank Sandu, Siva Sreedhar, Linda Chang, Lindsay Cohen, Andrea Cruz, Heidi R. Olson, Radhika Sreedhar, Kayeromi Gomez, Alberto Carrion

**Affiliations:** ^1^Department of Biochemistry and Molecular Genetics, University of Illinois Chicago, Chicago, IL, United States; ^2^Department of Chemical Engineering, University of Illinois Chicago, Chicago, IL, United States; ^3^Department of Family and Community Medicine, University of Illinois Chicago, Chicago, IL, United States; ^4^University of Illinois College of Pharmacy, Chicago, IL, United States; ^5^Department of Biological Sciences, University of Illinois Chicago, Chicago, IL, United States; ^6^Department of Pharmacy Practice, University of Illinois Chicago College of Pharmacy, Chicago, IL, United States; ^7^Department of Medicine, University of Illinois Chicago, Chicago, IL, United States; ^8^Department of Epidemiology and Biostatistics, University of Illinois Chicago, Chicago, IL, United States; ^9^Lifescape Community Services, Rockford, IL, United States

**Keywords:** social isolation, telecommunication, neighbor, student volunteers, social determinants of health, scalable program, loneliness, older adults

## Abstract

**Aim:** In this once-in-a-lifetime humanitarian crisis, what does it mean to be a good neighbor? It means that as a community, we must address loneliness and barriers to care faced by vulnerable populations such as older adults. We share an inexpensive longitudinal experiential service-learning program implemented by health professions and undergraduate student volunteers that aims to help alleviate loneliness in older adults while imparting meaningful experiences to volunteers.

**Intervention Design and Setting:** The 21st Century Good Neighbor Program is an observational cohort study of an experiential service-learning program started in May 2020, and this article shares the results collected after 1 year. This longitudinal, weekly phone call program was conducted in a single community setting in the Midwestern part of the United States. Older adults over the age of 60 served by a local community service agency (CSA) were invited to participate. Volunteers consisted of students 18 or older. Student volunteers made regular phone calls to a pair of older adults throughout the course of 1 year following standardized call scripts. The loneliness of the older adults was measured by volunteers using the 3-item UCLA Loneliness Assessment.

**Results:** 261 older adults were engaged in conversations with a volunteer. A total of 1,391 calls were accepted by older adults and the median length of a welcomed call was 11 min. The average baseline loneliness score was 4.156 ± 1.41 and the prevalence of social isolation was 19.5%. There was no significant change in the UCLA loneliness score in the first year of follow up. However, a majority of volunteers (88%) agreed or strongly agreed that the program had a positive impact on them. In addition, the program identified 257 issues older adults faced that required follow-up. The most prevalent concerns referred to the community service agency by volunteers were issues related to utilities, food and transportation access.

**Conclusion:** The 21st Century Good Neighbor Program is a unique intervention in which student volunteers and older adults paired by a community service agency forge relationships though a longitudinal phone call-based program. This easy-to-implement program provides another layer of support to identify and refer issues that impact social determinants of health. The added benefit of volunteer satisfaction in the setting of COVID 19 pandemic is heartening. We hope to continue to study the impact of this intervention on social isolation in this vulnerable population.

## Introduction

With the rise of the COVID-19 pandemic, social isolation has grown rapidly throughout the country (1). A particularly vulnerable demographic, those above the age of 50, have historically had the largest rates of social isolation before this pandemic (2). Social isolation can be determined by the size of the network a person possesses, as well as the frequency with which a person engages meaningfully with individuals in their network (3). Loneliness, a variable defined as the discrepancy between an individual's perceived social connections and desired social connections, is also frequently discussed in relation to social isolation (3, 4). Both are found to be dependent on geographic location, gender, age, and income (5–7).

Loneliness and social isolation are affected by neighborhood factors. One convergent mixed-methods study showed that adults who lived in more densely populated areas of a city experienced significantly less loneliness and social isolation, and a sense of community even when variables such as gender, socioeconomic status, and ethnicity were controlled for (6). It is also well-known that loneliness and social isolation can impact health. Loneliness has been associated with a higher risk for developing dementia, less physical activity, depressive symptoms, undernutrition, lack of life satisfaction, and higher risk of mortality (8–11).

This pandemic has been unprecedented in both the duration and magnitude of physical distancing measures implemented by the federal government, leaving many cut off from physical and beneficial social connections. The COVID-19 pandemic has exacerbated many deleterious effects linked to loneliness and social isolation—such as anxiety and depression—in many age groups (12, 13). Recent studies suggest that older adult groups are particularly susceptible to being adversely affected by the COVID-19 pandemic and have one of the highest risks of any age group for developing clinical depression, anxiety, and other disorders from social isolation (2, 3, 14, 15). It follows then that addressing loneliness and social isolation plays a vital role in ensuring the health of individuals, making it a key target for intervention, especially during the ongoing COVID-19 pandemic. This is true for students and young adults as well, although the impact is not as dramatic.

A literature search in the field of social gerontology reveals multiple ways in which older adults experience social connections. The framework characterizing the experience of social experiences in later life includes three areas: personal relationships, community connections, and social engagement (16). Studies suggest that preventive social services should target new contacts in developing opportunities for socialization among older adults through a framework called MODEL (Model of Depression and Loneliness). A systematic review on interventions targeting loneliness and social isolation among older adults suggests that new technologies and community-engaged arts are promising tools to combat the same (17).

In this descriptive study, we evaluate the longitudinal effects of using simple technology like telephone calls to foster social engagement and potentially reduce the social isolation among older adults (18–20). The 21st Century Good Neighbor Program is centered around student volunteers from a public university (University of Illinois Chicago) making phone calls to older adults who are served by a community services agency in IL. Calls made to older adults by student volunteers are outlined by standardized call scripts. The ongoing partnership with the community service agency helps provide a method to quickly address any issues reported by older adults. This phone-based intervention creates virtual spaces for meaningful longitudinal engagement of student volunteers with a “neighbor” providing much needed social connections during, and potentially after, the COVID-19 pandemic.

## Materials and Methods

### Recruitment of Participants

Older adults aged 60 years and over living within the 8 counties served by the community service agency (CSA) were invited to participate in this program. This was done by mailing a postcard informing them of the 21st Century Good Neighbor Program.

Students aged 18 years and older were invited to participate as volunteers in this service-learning program. These volunteers were recruited through multiple means including emails and student organizations in the colleges of pharmacy, medicine, engineering, liberal arts and sciences, applied health sciences, and public health.

### Intervention

The community service agency (CSA) paired student volunteers with their older adult clients in the community. During the first call, if the older adult consented to participate in the program, a trained volunteer continued the phone call using the program's standardized phone scripts (Supplementary Material).

### Phone Call Scripts

These scripts were created by student and faculty leaders at the inception of the program and provided electronically for student volunteers to fill out (provided in Supplementary Material). The scripts contain both explicit questions volunteers need to answer and provided tips for engaging in active conversations.

The 1st phone script served as an introductory conversational call about health and well-being, provided health information on COVID-19, and assisted participants in accessing resources such as meals and utility support. A copy of the call scripts can be found within the Supplementary Material.

The 2nd phone call script (used for calls conducted a week later), prompted the volunteer to enquire about the health of the older adult and complete an assessment on loneliness using the brief UCLA Loneliness Scale (21). A score of 5 or above on this scale suggests that the adult is experiencing social isolation. Volunteers were instructed to utilize the UCLA Loneliness Scale on the 2nd phone call because loneliness is a sensitive topic; volunteers used the first phone call to instead build a sense of trust with their older adult client.

The 3rd phone call script prompted the volunteer to assess the need for health and wellness services and other senior-related assistance programs available through the community service agency. The final scripted phone call continued to provide an opportunity for the established relationship to grow, and to help volunteers gain confidence in engaging conversation with older adults and learning from the older adults' experience about some social determinants of health.

**Figure 1:**
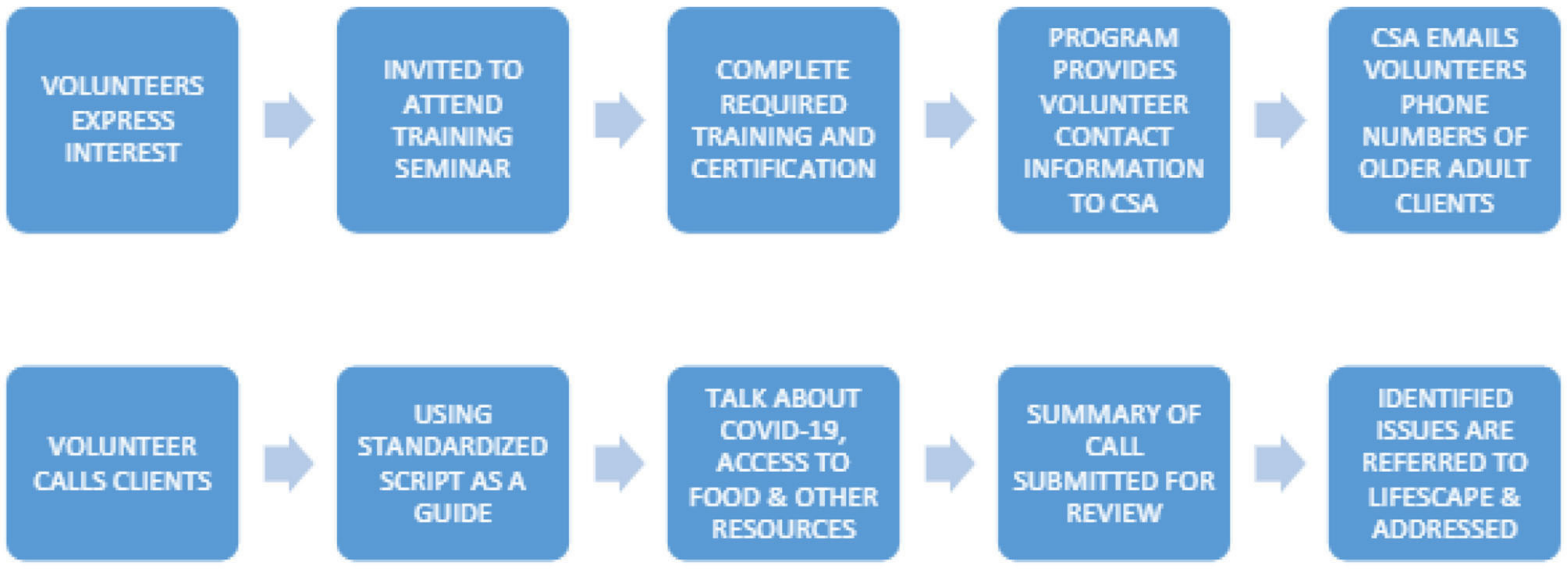
Flowchart outlining 21st Century Good Neighbor Program's intervention structure, steps taken by volunteers to engage in meaningful social interaction with older adults, and the methodology for collecting information.

After the third phone call no more scripts were used. However, students were prompted after each phone call, any identified social issues and client needs were documented by the volunteer and referred to the CSA to be addressed. A summary of this intervention procedure is captured below in Figure 1.

This program fostered partnerships between student volunteers and the CSA by identifying problems that clients have, and a pathway for follow-up on the identified problems. It also fostered a mutually beneficial longitudinal relationship between the volunteers and older adults.

### Statistical Analysis

An exemption waiver for IRB approval was granted by the UIC Rockford IRB to analyze the data obtained from the calls. The IRB approval number is 1713652-2. Descriptive statistics (e.g., frequencies, mean and standard deviation), were used to describe the demographics. Results are presented as mean ± standard deviation (SD) or as frequencies and percentages. The median with the interquartile range (IQR) were used to describe non-parametric data. An alpha of <0.05 was set as a level of statistical significance. All data analysis was conducted on Python 3.8.

## Results

1361 older adults were assigned to volunteers. 62% of the population identified themselves as female. 28% of the adults identified themselves as Black. 5% identified as Hispanic and the rest identified as White. The majority of the older adults, specifically 920 (67%), fell into the low-income category. 817 or 60% of these older adults lived by themselves, while 24% lived with one other person. Three percent of the older adults contacted were veterans.

Four hundred and eighty volunteers were in the inception cohort. Forty percent of the volunteers were undergraduate students at UIC, with the remaining volunteers attending various graduate programs at UIC–most commonly the Schools of Medicine, Pharmacy and Public Health at UIC. Approximately 276 (50%) of the initial enlisted volunteers were undergraduates, 88 (20%) were medical students, 57 (14%) were pharmacy students, and 59 (14%) were public health students.

**Figure 2:**
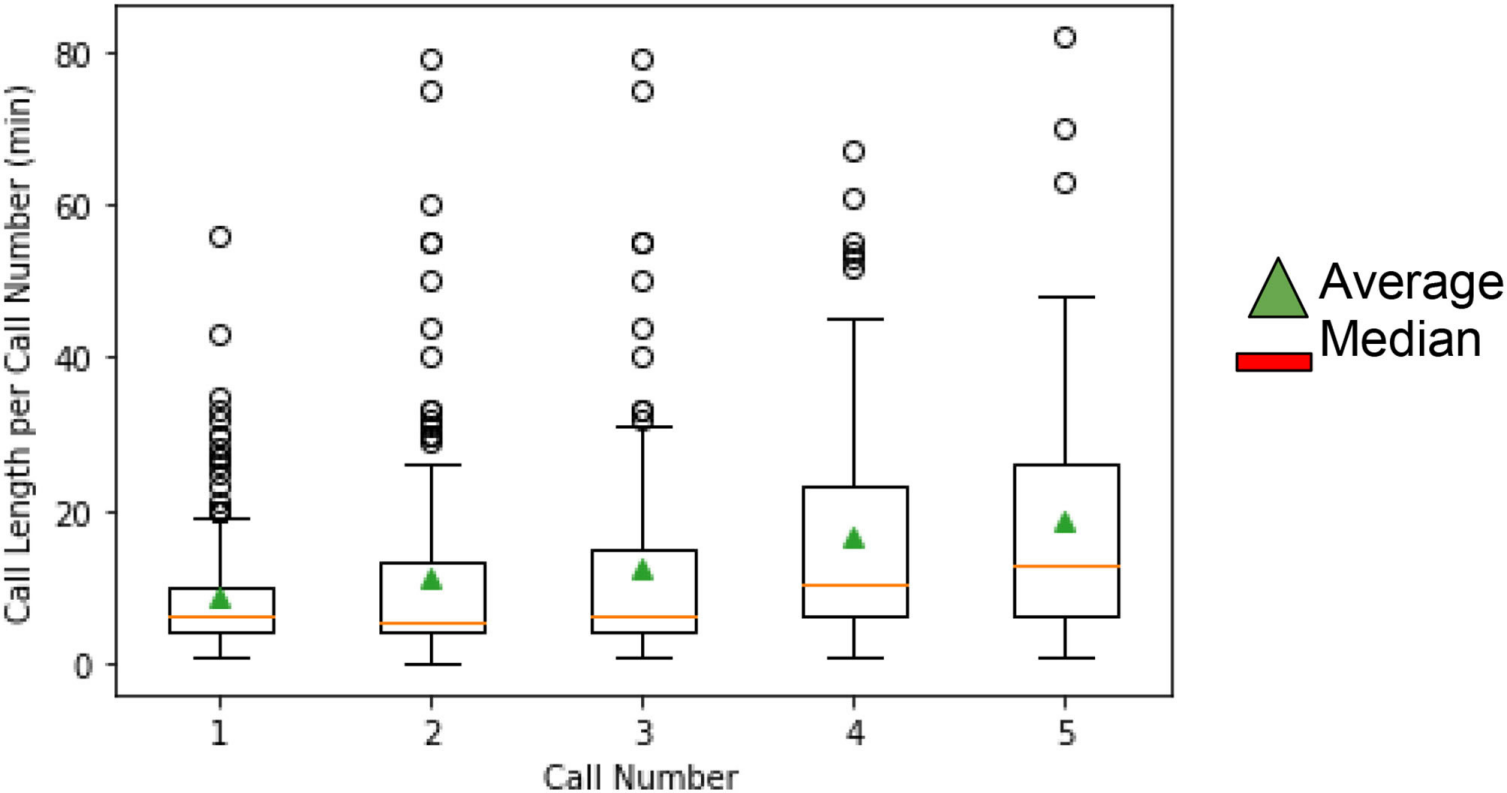
Call duration per call number. This figure depicts the descriptive statistics for the length of call in minutes per call number.

A total of 1,391 calls were welcomed and the median length of a welcomed call was 11 min IQR (18). The descriptive statistics for the duration of calls by call number is provided in Figure 2.

### UCLA Loneliness Assessment of Older Adults

The UCLA Loneliness assessments were initially assessed in 141 older adults, and the average baseline score was 4.156 ± 1.41. The initial score of the 84 adults who completed at least one follow-up UCLA questionnaire was 4.15 ± 1.41. This was similar to the score of 4.16 ± 1.00 for the 57 adults who did not complete the follow-up UCLA questionnaire. No significant relationship was found between the change in UCLA Loneliness Assessment score and either the duration or number of calls.

### Client Issues Requiring Follow Up

**Figure 3:**
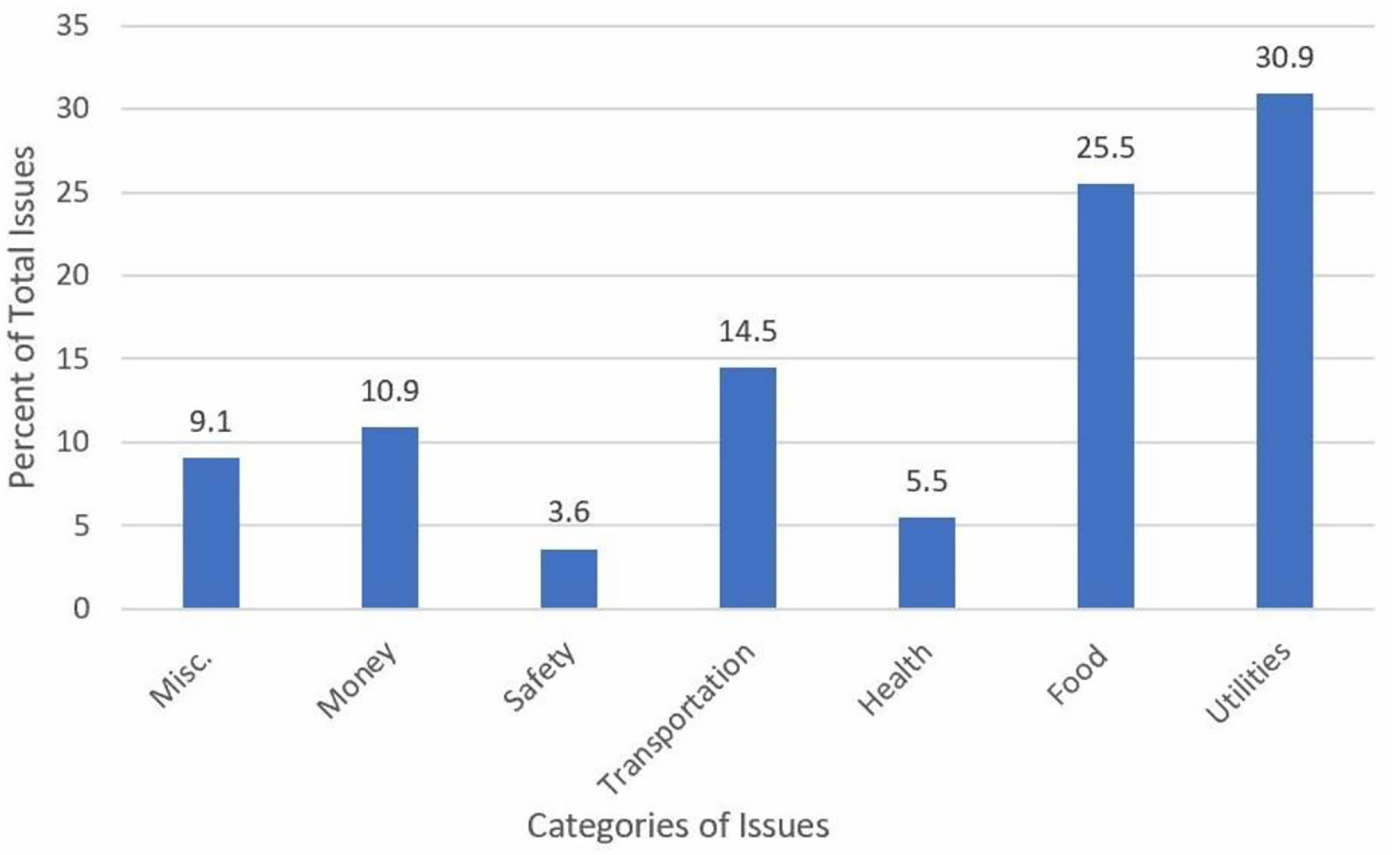
Categories of client issues that were referred for follow up.

Volunteers identified 257 issues requiring follow-up as a result of these calls. Of these, detailed information was provided for 132 issues. Access to necessities like food and utilities accounted for over 50% of these issues, followed by monetary issues and lack of access to transportation (see Figure 3).

### Volunteer Satisfaction

**Figure 4:**
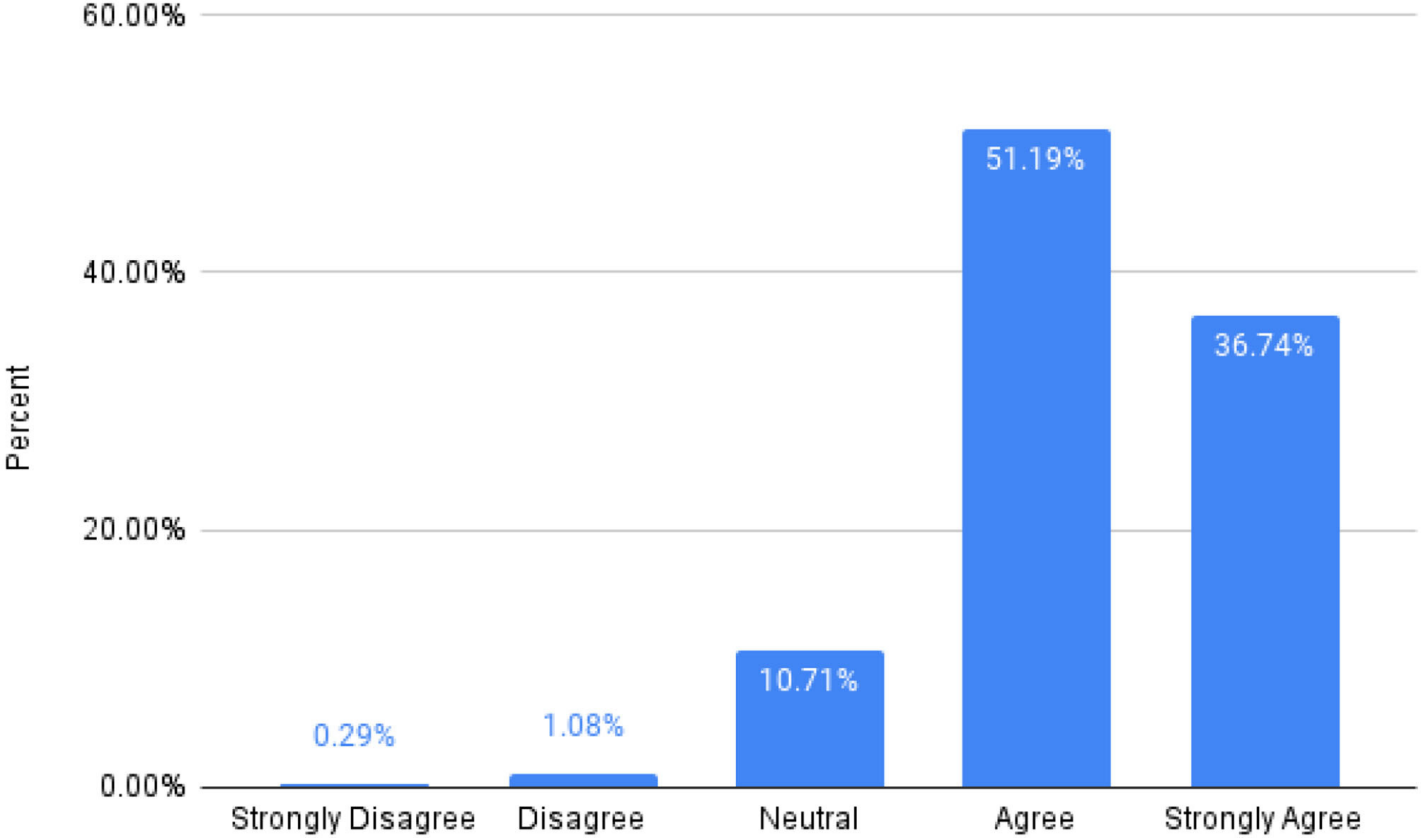
Percentage distribution of satisfaction scores in volunteers. This chart depicts the percentage of volunteers who agree that the program has made a significant impact on their life (administered through a volunteer satisfaction survey). Nearly 88% of the calls were reported to have a positive and meaningful impact on the volunteer's life, with <2% of calls resulting in a disagreement with the statement presented.

The impact of each call on the volunteer's life was measured based on responses to the statement “The call led to a renewed sense of meaning and purpose in my life.” The students were asked to rate their agreement with this statement on a five-point Likert scale: 1 (strongly disagree), 2 (disagree) 3 (neutral), 4 (agree), 5 (strongly agree). As shown in Figure 4, 88% of volunteers agreed with the aforementioned statement.

## Discussion

The purpose of this service-learning program was to mitigate the deleterious effects loneliness may have on community dwelling older adults by building lasting and meaningful social relationships with student volunteers. This intervention is novel in that it provided the opportunity for a longitudinal relationship between the volunteer and older adult. The collaboration with the aging agency helped address issues related to social determinants of health that affected older adults during the pandemic.

There has been an explosion of research showing the harm of social isolation to populations at-risk for depression, suicidal thoughts, or other indicators of an unhealthy mental or physical state (2). In light of these pressures, no change in loneliness score is important. Among older adults with no change in loneliness score, we noticed a trend towards significance in the relationship between increasing duration of calls and UCLA loneliness score. We will follow up to determine whether the investment of more time in fostering the relationship will help to combat this loneliness epidemic (see Figure 2).

Few studies have investigated the benefit volunteers gain from a meaningful relationship with older adults. In the setting of the pandemic, it is exciting to see the benefit volunteers experience from participation in this program.

One of the limitations of this study is that only 141 of all 261 adults completed the first UCLA questionnaire. Of the 141, only 84 completed the second UCLA questionnaire with an attrition rate of 40%. However, the program is ongoing and we hope to complete the remaining questionnaires.

As de-identified data was used, we could not account for missing data. To account for non-response bias, we compared the initial loneliness scores for the group that completed one questionnaire to that of the group who completed multiple questionnaires. The loneliness scores were similar.

Given that older adults participating in this program were already enrolled with a community service agency, they may already have been in need of aid, so it is likely that the data cannot be generalized to the population at large.

One randomized controlled trial of peer support using home visits and telephone calls, showed a statistically significant decrease in loneliness and increased resilience among older Chinese immigrants in Canada (22). Yet another program in Japan showed that interventions aimed at preventing social isolation in older adults were effective when they utilize existing community resources (23). The 21 Century Good Neighbor program is similar to these studies in that it is a volunteer-based phone call program using existing community resources by partnering with a community services agency.

In contrast to similar studies within the United States, volunteers in the program underwent mandatory training to actively engage in meaningful social interactions with older adults, referring older adults to the Community Service Agency, and reporting elder abuse (24). Our program is unique in that it is longitudinal; the same student continues to talk to the older adult on and off for a year, building valuable rapport.

When assessing volunteer satisfaction with the program, we used a single question (see Figure 4 legend) to ensure that our volunteers reflected on the benefit of their interactions with each call. Despite the social desirability bias that may come with this method of assessing satisfaction, this threat is inevitable in any intervention with a similar humanistic goal that assesses “satisfaction” among its volunteers or employees. To assess volunteer satisfaction, we used a short question, rather than a long, validated questionnaire. We felt that this would easily capture the desired information and also identify if volunteers experienced burnout.

We expect that using volunteers leads to selection bias, which exists for any similar intervention where the goal is to help another person. We see this “bias” as a strength of our volunteer-based intervention. Selection bias is also an issue as the older adults who agree to participate in the intervention may be different from the rest of the population.

In a time as challenging as this pandemic, obtaining real-time information on the well-being of the older adults is necessary and important. For instance, almost half the older adult clients reported lacking basic necessities for survival, such as insufficient food, water, electricity, etc. This program offers a novel method through which we can address in real-time problems older adults may be facing. The fact that these issues were unaddressed until adults brought them up to the student volunteers is of much concern. Many other older adults may silently be suffering from these issues and may not have family or friends to turn to.

We recognize that many of these results can also be due to confounding variables. We plan to explore the effects of gender, age, socioeconomic status, ethnicity, and income on loneliness and social isolation in the future.

The central focus of this paper is to outline the program's structure and its potential to cultivate relationships between volunteers and older adults. One limitation is that each volunteer older adult pair is unique and that results may not be very easily generalized. However, we have shared this approach with sister institutions in the Midwest and have been able to successfully implement similar programs. The generalizability of the data to beyond the Midwest and to non-English speaking cohorts of older adults is to be determined.

This paper provides the framework for this initiative, with the hope that institutions of higher learning can spearhead this in their own communities. This would provide a broad base for further evaluation of the effects of using simple measures like phone calls on limiting loneliness in the older adult populations throughout the country.

As the pandemic subsides in the future, further examination can be conducted using a hybrid approach of both telecommunication and in-person visits on loneliness levels of adults. Building meaningful social relationships, engaging in conversation about new topics, and maintaining mental health in older adults must be approached with an open mindset and multidisciplinary approach. This entails innovating resource-efficient interventions that are accessible to older adults and utilizing volunteers ready to learn how to actively engage with older adults and help; this program represents a step forward in protecting mental health during the 2020 COVID-19 pandemic (25–27).

In conclusion, the 21st Century Neighbor Good Neighbor program was established to fight the rise of loneliness during the COVID-19 pandemic. Through a volunteer base that made phone calls to their assigned older adults, a longitudinal connection was formed that expanded the social networks of elderly individuals in Illinois. A positive effect of the program was seen among volunteers as most calls led to a renewed meaning and purpose in the lives of volunteers.

This program is by no means a causal remedy for loneliness or depression. After all, no one program is. One of the most outstanding aspects of this program, in addition to its longitudinal nature, is its easily implementable and scalable structure that shows promising results for reducing loneliness of older adults. The investment needed is minimal and it can be easily adopted by other universities. In turn, this can lead to not only less lonely older adults, but also unprecedented levels of student engagement and responsibility. This reasoning lends itself to the potential ubiquity and demonstrated utility of this program; it follows that from the observed results, similarly implemented and organized programs in other undergraduate and graduate institutions could lead to a remarkable change in loneliness experienced by older adults and increased sense of self-worth in student volunteers. This may allow for the development of more creative, efficient multidisciplinary solutions to help improve the quality of older adults' lives (10).

## Data Availability Statement

The raw data supporting the conclusions of this article will be made available by the authors, without undue reservation.

## Ethics Statement

The studies involving human participants were reviewed and approved by IRB Administrative Body–(Exempt). Written informed consent for participation was not required for this study in accordance with the national legislation and the institutional requirements.

## Author Contributions

SSr and SSa designed and conducted the statistical tests used on Python. ACr helped construct the call scripts necessary for data collection. SSr, SSa, LCo, and ACr were involved in writing the manuscript. LCh, RS, and HO oversaw the progress of the manuscript and created the 21st Century Good Neighbor program, KG oversaw the data analysis and ACa provided part of the demographic data used. All authors contributed to the article and approved the submitted version.

## Conflict of Interest

The authors declare that the research was conducted in the absence of any commercial or financial relationships that could be construed as a potential conflict of interest.

## Publisher's Note

All claims expressed in this article are solely those of the authors and do not necessarily represent those of their affiliated organizations, or those of the publisher, the editors and the reviewers. Any product that may be evaluated in this article, or claim that may be made by its manufacturer, is not guaranteed or endorsed by the publisher.

## Supplementary Material

Supplementary Material The four call scripts used in this intervention.
