# Supplementary material for: 21st Century Good Neighbor Program: An Easily Generalizable Program to Reduce Social Isolation in Older Adults
Source: Front Public Health. 2021 Dec 20;9:766706. doi: 10.3389/fpubh.2021.766706 (PMC8721124; doi:10.3389/fpubh.2021.766706)
Supplement: Supplementary Material — The four call scripts used in this intervention. [file Data_Sheet_1.pdf]

Client Name: ______________________ Lifescape ID#: _____________ Date of Call: _____________ **21st**

Century Good Neighbor – 1st Phone Call Script

Goals:

- Check in consistently with community members who might be more vulnerable to COVID-19. • Assist with access to services, as indicated.
- Provide accurate information on COVID-19.

General Guidance:

- It is important that you are calm and provide accurate information, because many people are scared. • Report back to agency and a caseworker will review case. We are not asking you to manage individual needs.
- Creating a regular cadence for calls is reassuring to those being called. If you can call the same person on the same day of the week, that is ideal.

Call Script

INTRO

Hi (*Client name*), My name is _____________ and I'm from the ____(college/university and program name)___ *(i.e. University of Illinois at Chicago, College of Pharmacy*). I am a (medical, pharmacy, nursing, etc.) student in my _____________(first, second, third, fourth, etc.) year of school. The College is collaborating with Lifescape in reaching out to their clients during this time to see how they are doing and if they need anything.

Is it okay if I ask you a few questions about how you are doing? Yes ☐ No ☐

If NO →(Assess if calling back another time is better for resident. If they do not want to have a conversation, please provide the ***Lifescape phone #*** 1-815-963-1609 or 1-800-779-1189 and thank them for their time. Complete contact report.)

Better time to call back: ____

If YES:

1. I'm going to go through a list of symptoms. Let me know if you are experiencing any of them today.
   a. **Fever or chills** Yes ☐ No ☐
   b. **Cough** Yes ☐ No ☐
   c. **Shortness of breath** Yes ☐ No ☐
   d. Congestion (nose, lungs) Yes ☐ No ☐
   e. Sore throat Yes ☐ No ☐
   f. Body aches Yes ☐ No ☐

g. Unusual fatigue Yes ☐ No ☐

-If NO to all symptoms: I'm glad you don't have any symptoms right now.

-If YES to any symptoms: I'm sorry to hear that.

Do you think any of your symptoms are severe?

i. **YES symptoms are severe**:

- Instruct client to call 911

ii. **NO, symptoms are not severe:** It is *really important* that you stay inside. We don't want anyone who is sick or starting to feel sick to go outside. When we get done speaking, you should call your primary care provider.

- If client doesn't have a primary care provider, ask client to call Lifescape 1-815-963-1609 or 1-800-779-1189 for help finding a provider and/or addressing health insurance, transportation or other issues.

2. Do you have any concerns about COVID-19? Yes ☐ No ☐ If NO, go to question 3.

3. We want to make sure that everyone has the food that they need.

- Do you have enough food for today and tomorrow? Yes ☐ No ☐

a. **If YES**: Great!

b. **If NO:** Have the senior call *Lifescape at* 1-815-963-1609 or 1-800-779-1189 to discuss Meals on Wheels or call the Illinois Senior Helpline at 1-800-252-8966

i. If needed, here is a resource to find local food pantries

- Enter client zip code in this website to find nearby locations: https://solvehungertoday.org/get-help/where-to-get-food/

ii. If needed, tell them that some stores have dedicated shopping hours for senior citizens.

4. Are your lights and electricity on? Yes ☐ No ☐ **If YES**: Great!

If NO: Instruct client to contact **Lifescape #** 1-815-963-1609 or 1-800-779-1189

5. Do you live alone? Yes ☐ No ☐ **If NO:** Do you mind if I ask who lives with you? Spouse ☐ Child ☐ Other ☐ **If YES:** Do you have any family members or friends who support you in any way? Yes ☐ No ☐

If YES: That's great. It's important to keep in touch with family at this time.

If NO: Well, we will start checking in on you more regularly.

Summary:

1. Thank you for taking the time to talk. Is there anything else that you're worried about right now? ____
2. Can I call you again in a week? Evening or weekend hours are better for me, is there a time that works better for you? ____

-----------------------END SCRIPT-------------------------------

Now that the call is completed, please complete the Contact Report.

************Information about COVID-19 – Could be included in

conversation*********

We want to make sure that you know a few things about the Coronavirus, because we know that there is a lot of misinformation out there.

- We are trying to stop the spread of Coronavirus by staying home and leaving the house only for essential things, like groceries, laundry, etc. It is okay to take a walk, but this is not the time for socializing outside or at someone else's home.
  - If you are over 60 and/or have any medical condition that affects your immune system, like diabetes, cancer, lung problems, etc., then we really want you to stay home AND stay away from others who might be sick.
- If you begin to have any symptoms, like the ones we talked about, please call your doctor immediately, especially if you are over 60 and/or have any other medical conditions.
- Coronavirus is spread through droplets that come from a person's mouth or nose (from coughing or sneezing) and get into another person's eyes, nose, or mouth. • It is most often passed between people who are in close contact with each other – like people who live together.
  - If someone who lives with you is sick or feeling sick, try to keep your distance and make sure that you are washing your hands and surfaces in the house. • YOUR best defense against the Coronavirus is to:
  - Wash your hands often with soap and water for at least 20 seconds, especially after blowing your nose, coughing, or sneezing, or having been in a public place.
    - If soap and water are not available, use a hand sanitizer that contains at least 60% alcohol.
    - When hands are visibly dirty, always wash with soap and water.
  - To the extent possible, avoid touching high-touch surfaces in public places – elevator buttons, door handles, handrails, handshaking with people, etc.
    - Use a tissue or your sleeve to cover your hand or finger if you must touch something.
    - Wash your hands or use hand sanitizer after touching surfaces in public places.
  - Avoid touching your face, nose, eyes, etc.

Clean and disinfect- Per CDC cleaning instruction:

- **Clean AND disinfect frequently touched surfaces daily**. This includes tables, doorknobs, light switches, countertops, handles, desks, phones, keyboards, toilets, faucets, and sinks.
- **If surfaces are dirty, clean them:** Use detergent or soap and water prior to disinfection.

To disinfect:

Most common EPA-registered household disinfectants will work. Use disinfectants appropriate for the surface.

Options include:

- **Diluting your household bleach.**

  To make a bleach solution, mix:
  - 5 tablespoons (1/3rd cup) bleach per gallon of water

    OR
  - 4 teaspoons bleach per quart of water

  Follow manufacturer's instructions for application and proper ventilation. Check to ensure the product is not past its expiration date. Never mix household bleach with ammonia or any other cleanser. Unexpired household bleach will be effective against coronaviruses when properly diluted.
- **Alcohol solutions.**

  Ensure solution has at least 70% alcohol.
- **Other common EPA-registered household disinfectants.**

  Products with EPA-approved emerging viral pathogens pdf icon[7 pages]external icon claims are expected to be effective against COVID-19 based on data for harder to kill viruses. Follow the manufacturer's instructions for all cleaning and disinfection products (e.g., concentration, application method and contact time, etc.).
  - Clean and disinfect your home to remove germs: practice routine cleaning of frequently touched surfaces (for example: tables, doorknobs, light switches, handles, desks, toilets, faucets, sinks & cell phones).
  - Avoid close contact with people who are sick.

General Resources

Coronavirus Page: https://rockfordil.gov/preparing-for-covid-19/

CDPH Guidance for Seniors:

https://www2.illinois.gov/aging/coronavirus/Pages/default.aspx **Illinois Coronavirus**

Response: https://coronavirus.illinois.gov/s/

Food Resources for Seniors:
https://lifescapeservices.org/services/nutrition/
https://solvehungertoday.org/coronavirus/

Non-Emergency Police #s Winnebago County, IL:
Rockford 1-815-966-2900
Outside Rockford 1-815-282-2600

Program Partners

UNIVERSITY OF ILLINOIS CHICAGO
College of Pharmacy
CHICAGO • ROCKFORD

UNIVERSITY OF ILLINOIS
COLLEGE OF MEDICINE | ROCKFORD
