# Supplementary material for: 21st Century Good Neighbor Program: An Easily Generalizable Program to Reduce Social Isolation in Older Adults
Source: Front Public Health. 2021 Dec 20;9:766706. doi: 10.3389/fpubh.2021.766706 (PMC8721124; doi:10.3389/fpubh.2021.766706)
Supplement: Supplementary file 2 [file Data_Sheet_2.pdf]

Client Name: ____________ Lifescape ID#: ________ Date of Call: ______

## 21st Century Good Neighbor™ – 2nd Phone Call Script

**Call Script**

Hi (Client Name), this is            (name)  from the            (college/university and program name). We talked last week about concerns regarding COVID 19 and            .

I'm calling today to follow up with you and see how you're doing.
Have you been doing well since the last time we spoke? Yes ☐ No ☐

If **YES**, go to question 2.
If **NO**, go to question 1 included below.

1. I'm going to go through a list of symptoms. Let me know if you are experiencing any of them today.

| | | |
|---|---|---|
| **Fever or chills** | Yes ☐ | No ☐ |
| **Cough** | Yes ☐ | No ☐ |
| **Shortness of breath** | Yes ☐ | No ☐ |
| Congestion (nose, lungs) | Yes ☐ | No ☐ |
| Sore throat | Yes ☐ | No ☐ |
| Body aches | Yes ☐ | No ☐ |
| Unusual fatigue | Yes ☐ | No ☐ |

If **NO** to all symptoms: I'm glad you don't have any symptoms right now.
If **YES** to any symptoms: I'm sorry to hear that.

Do you think any of your symptoms are severe? Yes ☐ No ☐

**YES, symptoms are severe**: Instruct client to call 911
**NO, symptoms are not severe:** It is *really important* that you stay inside. We don't want anyone who is sick or starting to feel sick to go outside. When we get done speaking, you should call your primary care provider.

1. If client doesn't have a primary care provider, refer to **Lifescape** (**1-815-963-1609 or 1-800-779-1189)** for help finding a provider and/or addressing health insurance, transportation or other issues.

2. Because of the social distancing that we are all experiencing would it be ok for me to ask you a few questions? Yes ☐ No ☐

If **YES**: Ask your client these 3 questions as written and in the listed order.
*(This is a validated UCLA questionnaire so **using it as written is essential**. A score of 6 or higher indicates loneliness.)*

| | | | |
|---|---|---|---|
| How often do you feel that you lack companionship? | Hardly Ever ☐<br>*1 point* | Some of the Time ☐<br>*2 points* | Often ☐<br>*3 points* |
| How often do you feel left out? | Hardly Ever ☐<br>*1 point* | Some of the Time ☐<br>*2 points* | Often ☐<br>*3 points* |
| How often do you feel isolated from others? | Hardly Ever ☐<br>*1 point* | Some of the Time ☐<br>*2 points* | Often ☐<br>*3 points* |

*If the client responds "**often**" for any of the above questions:*
I hear that you feel "(repeat an area that they score often)". I hope that our phone calls can help you feel less "(repeat an area that they score often)".

3. We want to make sure that everyone has the food that they need.
   - Do you have enough food for today and tomorrow? Yes ☐ No ☐

     If **YES**: Great!
     If **NO**: Have the senior call ***Lifescape* at (1-815-963-1609 or 1-800-779-1189)** to discuss Meals on Wheels or call the **Illinois Senior Helpline at (1-800-252-8966).**

     i. If needed, here is a resource to find local food pantries
        - Enter client zip code in this website to find nearby locations: https://solvehungertoday.org/get-help/where-to-get-food/

     ii. If needed, tell them some stores have dedicated shopping hours for senior citizens

4. Are your lights and electricity on? Yes ☐ No ☐

   **If YES**: Great!
   **If NO:** Instruct client to contact **Lifescape** at **(1-815-963-1609 or 1-800-779-1189).**

**Summary:**
1. Thank you for taking the time to talk. Is there anything else that you're worried about right now?
2. If the resident has further concerns, please provide the ***Lifescape*** phone number **(1-815-963-1609; 1-800-779-1189).**
3. Would it be ok if I called you again in a week?

4. End conversation in a way you are most comfortable with (*Have a good night. Have a good weekend. Stay safe.*).

**END SCRIPT**

**Now that the call is completed, please complete the Contact Report.**

**General Resources**

**Coronavirus Page:** https://rockfordil.gov/preparing-for-covid-19/

**CDPH Guidance for Seniors:** https://www2.illinois.gov/aging/coronavirus/Pages/default.aspx

**Illinois Coronavirus Response:** https://coronavirus.illinois.gov/s/

**Food Resources for Seniors:**
https://lifescapeservices.org/services/nutrition/
https://solvehungertoday.org/coronavirus/

**Non-Emergency Police #s Winnebago County, IL:**

| | |
|---|---|
| Rockford | 1-815-966-2900 |
| Outside Rockford | 1-815-282-2600 |

**Elder Abuse, Neglect, Exploitation & Self-Neglect Hotline**
24-hour Adult Protective Services Hotline: 1-866-800-1409, 1-888-206-1327 (TTY)

**Program Partners**

UIC UNIVERSITY OF ILLINOIS CHICAGO
College of Pharmacy
CHICAGO • ROCKFORD

UIC UNIVERSITY OF ILLINOIS
COLLEGE OF MEDICINE | ROCKFORD
