# Supplementary material for: 21st Century Good Neighbor Program: An Easily Generalizable Program to Reduce Social Isolation in Older Adults
Source: Front Public Health. 2021 Dec 20;9:766706. doi: 10.3389/fpubh.2021.766706 (PMC8721124; doi:10.3389/fpubh.2021.766706)
Supplement: Supplementary file 3 [file Data_Sheet_3.pdf]

Client Name: Lifescape ID#: Date of Call:

21st Century Good Neighbor™ – 3rd Phone Call Script

Goals:

- Check in consistently with community members who might be more vulnerable to COVID-19.
- Assist with access to services, as indicated.
- Provide accurate information on COVID-19.

General Guidance:

- It is important that you are calm and provide accurate information, because many people are scared.
- Report back to agency and a caseworker will review case. We are not asking you to manage individual needs.
- Creating a regular cadence for calls is reassuring to those being called. If you can call the same person on the same day of the week, that is ideal.

Call Script

Hi (Client Name), this is (name) from the (college/university and program name). We talked last week about concerns regarding COVID 19 and

I'm calling today to follow up with you and see how you're doing.
Have you been doing well since the last time we spoke? Yes ☐ No ☐

If **YES**, go to question icebreaker questions.
If **NO**, go to question 1 included below.

1. I'm going to go through a list of symptoms. Let me know if you are experiencing any of them today.

**Fever or chills**	Yes ☐	No ☐
**Cough**	Yes ☐	No ☐
**Shortness of breath**	Yes ☐	No ☐
Congestion (nose, lungs)	Yes ☐	No ☐
Sore throat	Yes ☐	No ☐
Body aches	Yes ☐	No ☐

Unusual fatigue Yes ☐ No ☐

If **NO** to all symptoms: I'm glad you don't have any symptoms right now.
If **YES** to any symptoms: I'm sorry to hear that.

Do you think any of your symptoms are severe? Yes ☐ No ☐

YES symptoms are severe:

- Instruct client to call 911

NO, symptoms are not severe: It is *really important* that you stay inside. We don't want anyone who is sick or starting to feel sick to go outside. When we get done speaking, you should call your primary care provider.

- If client doesn't have a primary care provider, refer to **Lifescape** at **(1-815-963-1609 or 1-800-779-1189)** for help finding a provider and/or addressing health insurance, transportation or other issues.

Icebreaker questions

Chose a few of the questions below (or some of your own) to ask your client to help develop a rapport. (Pick questions that you can also relate to)

- What is your favorite sport?
- What is your favorite movie?
- Do you collect anything?
- Do you have any pets?
- What do you do for fun?
- Who is your hero?
- Are you a veteran?
- Are you retired? What did you used to do?
- What are you currently reading?
- What is your favorite television show?
- What does your perfect breakfast look like?
- Do you have any children? Grandchildren?
- What superpower would you like to have?
- What would the title of your autobiography be?
- What is the most memorable vacation you've ever taken?
- What is a skill you learned when you were young that you still use today?
- If you could have dinner with anyone, living or dead, who would it be?

Icebreaker Notes:

2. Because of the social distancing that we are all experiencing would it be ok for me to ask you a few questions? Yes ☐ No ☐

If **YES**: *Ask your client these 3 questions as written and in the listed order. (This is a validated UCLA questionnaire so* ***using it as written is essential****. A score of 6 or higher indicates loneliness)*

How often do you feel that you lack companionship?	Hardly Ever ☐ *1 point*	Some of the Time ☐ *2 points*	Often ☐ *3 points*
How often do you feel left out?	Hardly Ever ☐ *1 point*	Some of the Time ☐ *2 points*	Often ☐ *3 points*
How often do you feel isolated from others?	Hardly Ever ☐ *1 point*	Some of the Time ☐ *2 points*	Often ☐ *3 points*

*If the client responds "****often****" for any of the above questions:*
I hear that you feel "(repeat an area that they score often)". I hope that our phone calls can help you feel less "(repeat an area that they score often)". I look forward to talking with you next week.

Summary:

1. **Lifescape** is a good resource and is always available to help you if needed. They can be reached at (**1-815-963-1609; toll free 1-800-779-1189).**
2. Would it be ok if I called you again in a week? Yes ☐ No ☐
3. *End conversation in a way you are most comfortable with* - Have a good night. Have a good weekend. Stay safe.)

END SCRIPT

Now that the call is completed, please complete the Contact Report.

General Resources

Coronavirus Page: https://rockfordil.gov/preparing-for-covid-19/

CDPH Guidance for Seniors: https://www2.illinois.gov/aging/coronavirus/Pages/default.aspx

Illinois Coronavirus Response: https://coronavirus.illinois.gov/s/

Food Resources for Seniors:
https://lifescapeservices.org/services/nutrition/
https://solvehungertoday.org/coronavirus/

Non-Emergency Police #s Winnebago County, IL:

Rockford	1-815-966-2900
Outside Rockford	1-815-282-2600

Elder Abuse, Neglect, Exploitation & Self-Neglect Hotline
24-hour Adult Protective Services Hotline: 1-866-800-1409, 1-888-206-1327 (TTY)

Program Partners

UIC UNIVERSITY OF ILLINOIS CHICAGO
College of Pharmacy
CHICAGO • ROCKFORD

UIC UNIVERSITY OF ILLINOIS
COLLEGE OF MEDICINE | ROCKFORD
