# Supplementary material for: 21st Century Good Neighbor Program: An Easily Generalizable Program to Reduce Social Isolation in Older Adults
Source: Front Public Health. 2021 Dec 20;9:766706. doi: 10.3389/fpubh.2021.766706 (PMC8721124; doi:10.3389/fpubh.2021.766706)
Supplement: Supplementary file 4 [file Data_Sheet_4.pdf]

Client Name: Lifescape ID#: Date of Call:

21st Century Good Neighbor™ – Continuing Call Guide

Goals:

- Check in consistently with community members who might be more vulnerable to COVID-19.
- Assist with access to services, as indicated.
- Provide accurate information on COVID-19.

Call Script

Hi (Client Name), this is (name) from the (college/university and program name). We talked last week about concerns regarding COVID 19 and .

As you continue to build relationship with your client(s), here are a few tips to remember:

- Your purpose is to raise morale, and to help your new friend feel connected and hopeful.
- Focus on your conversation; turn off any background noise like tv, music etc.
- Keep the conversation positive. Sometimes just asking "How are you?" can lead to negative answers and a conversation about aches & pains or troubles. Try opening with, "What's new with you today?" or "It's so good to talk with you today."
- Ask about previously mentioned things such as hobbies, grandchildren or things they enjoy. Look back to the Icebreaker **Questions for Older Adults** for ideas if conversation does not come easily.
- Try to ask open-ended questions, which cannot be answered by yes, no, or one-word answers. Opening phrases could be "tell me about" or "what do you feel/think about".
- Remember to keep the conversation focused on the client and their interests.
- Be respectful, even if you don't agree with their opinion. Don't disregard their ideas; remember they have walked a different road than you have.
- Understand that the client may tell you the same thing more than once, or not remember what you talked about in the last conversation. Be sure not to condescend or belittle.
- Avoid giving advice, unless you are specifically asked.
- Refer back to **Lifescape Community Services at (815) 963-1609** for resources if necessary.
- Share happy stories, good news.
- End the call by reminding them that you will be calling again, give them something to look forward to.

Topic(s) discussed during this call

General Resources

Coronavirus Page: https://rockfordil.gov/preparing-for-covid-19/

CDPH Guidance for Seniors: https://www2.illinois.gov/aging/coronavirus/Pages/default.aspx

Illinois Coronavirus Response: https://coronavirus.illinois.gov/s/

Food Resources for Seniors:
https://lifescapeservices.org/services/nutrition/
https://solvehungertoday.org/coronavirus/

Non-Emergency Police #s Winnebago County, IL:
Rockford 1-815-966-2900
Outside Rockford 1-815-282-2600

Elder Abuse, Neglect, Exploitation & Self-Neglect Hotline
24-hour Adult Protective Services Hotline: 1-866-800-1409, 1-888-206-1327 (TTY)

Program Partners

UIC UNIVERSITY OF ILLINOIS CHICAGO
College of Pharmacy
CHICAGO • ROCKFORD

UIC UNIVERSITY OF ILLINOIS
COLLEGE OF MEDICINE | ROCKFORD
